# Supplementary material for: Research of multi-label text classification based on label attention and correlation networks
Source: PLoS One. 2024 Sep 30;19(9):e0311305. doi: 10.1371/journal.pone.0311305 (PMC11441674; doi:10.1371/journal.pone.0311305)
Supplement: Attachment — Submitted filename: response_to_reviewsers.docx [file pone.0311305.s001.docx]

Research of Multi-Label Text Classification based on Label Attention and Correlation Networks

**Rseply to Reviewer 1:**

1- Proofread the manuscript carefully to eliminate any grammatical errors or typos and ensure clarity and coherence in writing. Additionally, adhere to the formatting and style guidelines specified by the target journal or publication venue to enhance the professionalism of the manuscript.

Reply:

| Full text | Corrections in various sections  For example: downweight → down weight To summary → In summary  Modify the structure style according to the target journal. For example, change the title of section 2 from Multi-Label Text Classification based on Label Attention and Correlation Networks to Method, and change the title of section 3 from Experimental Section to Results. |
| --- | --- |

2- The abstract section is fragile. Please re-write an abstract section, explain the result obtained and contribution, improve a proposed method, etc. Please delete unnecessary information.

Reply:

| Abstract | Rewrite the abstract section. |
| --- | --- |

3- The authors should clearly state the limitations of the proposed method in other real applications.

Reply:

| 4 Limitations | Add the limitations of the proposed method. For example, the correlation network based solely on the label distribution may not capture the complex relationship between labels well. |
| --- | --- |

4- I suggest the authors add a table at the end of the literature review and compare the reviewed papers to clarify the research gap better.

Reply:

| 1 Introduction paragraph 5 | Add a table to compare the reviewed papers. |
| --- | --- |

5- Please write your contribution to this paper in the Introduction section.

Reply:

| 1 Introduction paragraph 7 (penultimate paragraph) | Write the main three contributions to this paper at the penultimate paragraph of the Introduction section. |
| --- | --- |

6- Please draw a flowchart of the proposed method.

Reply:

| 2 Methods paragraph 1 | Add a flowchart of the proposed method. |
| --- | --- |

7- How did the authors set parameters for their proposed algorithm? Please make sensitivities of these parameters to the performance of their proposed algorithm!

Reply:

| 3.3 Parameter Configuration paragraph 1-2 | As described in Section 3.3 Parameter Configuration, authors first selected a set of default parameters and then adjusted these parameters by experimenting with different parameter values to get the best performance. |
| --- | --- |
| 3.6.3 Comparison of Classification results, the last paragraph | Add the analysis of the impact of varying the selection parameter  on model performance. |

8- Incorporating relevant and recent academic sources could strengthen your paper's validity and give readers more context and background. a hybrid model based on convolutional neural network and long short-term memory for multi-label text classification, an automatic text summarization based on valuable sentences selection.

Reply:

| 1 Introduction paragraph 1-2 | Add literatures [6] an automatic text summarization based on valuable sentences selection and [12] a hybrid model based on convolutional neural network and long short-term memory for multi-label text classification. |
| --- | --- |

9- Expand the critical results in the conclusion. Focus on the main developments in the finale. Also, write the main contributions in the conclusion.

Reply:

| 4 Conclusions and Future Work paragraph 2 | Add the critical results. |
| --- | --- |
| 4 Conclusions and Future Work paragraph 1 | Simplify the content to focus on the main developments of the proposed method. |
| 4 Conclusions and Future Work paragraph 1 | Write the main contributions of the proposed method in the paper. |

10- Numerical results are good enough, but more explanations are required to analyze each figure presented.

Reply:

| 3.6.1 Comparison of Text Representation paragraph 2 | Add a more detailed description of the experimental data results shown in Figure 2. |
| --- | --- |
| 3.6.2 Comparison of Label Co-occurrence paragraph 2-3 | Add a more detailed description of the experimental data results shown in Figure 3 and Figure 4. |
| 3.6.3 Comparison of Classification results paragraph 4 and paragraph 7 | Add a more detailed description of the experimental data results shown in Figure 5 and Figure 6. |

11- Please Change the “conclusion” section to “ Conclusion and Future Work” and write future work.

Reply:

| 4.Conclusions and Future Work paragraph 3 | Change the title of section 4 from Conclusions to Conclusion and Future Work and write the future work from what aspects to do. |
| --- | --- |

12- All figures are of low quality, so please improve all of them.

Reply:

| All figures | All figures were improved by converting to vectors or adjusting the resolution. |
| --- | --- |

**Reply to Reviewer 2:**

1- Please include the full equation of the evaluation metrics.

Reply:

| 3.4. Evaluation Metrics | Add the full equation of the evaluation metrics. |
| --- | --- |

2- Have you considered the overlapping between labels?

| 4. Conclusions and Future Work paragraph 3 | We have not yet considered the overlap between labels in the current study, but we will consider it as a future work to extend the correlation network based on label distribution, for example, using the graph neural network to learn the internal relations of labels and handle increasingly complex structural relationships between labels in the real world, such as inclusion and overlap. |
| --- | --- |

Reply:

3- Was the proposed method suitable for addressing the problem on all datasets? If so, why? Were all datasets imbalanced? If so, which one was the hardest to balance?

Reply:

| 3.5 Analysis of Model Comparison paragraph 2-3 | Yes, the proposed method was suitable for addressing the problem on all datasets because the method considers document content, label correlation, and label imbalance comprehensively. It uses the label attention mechanism to obtain the text representation of a specific label, uses the distribution-based relevance network to introduce label correlation, and optimizes for label imbalance, which can get the most and sub-optimal results on Micro-F1, P@k and NDCG@k compared with other methods. The detailed presentation and experimental data are shown in Table 3 and Table 4 of paragraph 2-3 of Section 3.5 on page 18-19. |
| --- | --- |
| 3.6.3 Comparison of classification results paragraph 1-5 | Yes, all four datasets have varying degrees of imbalance between and within labels as described in paragraph 1-5 of Section 3.6.3 on page 22-23. |
| 3.6.3 Comparison of classification results paragraph 1 | The RCV1-v2 dataset was the hardest to balance due to the long-tail phenomenon as described in paragraph 1 of Section 3.6.3 on page 22. |

4- Please add critical analysis at the end of the previous studies to demonstrate the main motivation of the current study.

Reply:

| 1 Introduction paragraph 5 | Add critical analysis of the previous studies from aspects of text representation, label correlation and data imbalance in paragraph 5 of Section 1 on page 2. |
| --- | --- |
